# Performance of a five category front-of-pack labelling system – the 5-colour nutrition label – to differentiate nutritional quality of breakfast cereals in France

**DOI:** 10.1186/s12889-015-1522-y

**Published:** 2015-02-25

**Authors:** Chantal Julia, Emmanuelle Kesse-Guyot, Pauline Ducrot, Sandrine Péneau, Mathilde Touvier, Caroline Méjean, Serge Hercberg

**Affiliations:** Université Paris 13, Equipe de Recherche en Epidémiologie Nutritionnelle (EREN), Centre de Recherche en Epidémiologie et Statistiques, Inserm (U1153), Inra(U1125), Cnam, COMUE Sorbonne Paris Cité, 93017 Bobigny, France; Hôpital Avicenne (AP-HP), Département de Santé Publique, 93017 Bobigny, France

**Keywords:** Nutritional quality, Nutrient profiling systems, Reformulation, Nutritional labelling, Breakfast cereals

## Abstract

**Background:**

Breakfast cereals exhibit a wide variability in nutritional quality, and differences are not easily grasped by consumers. A simplified nutritional information system might contribute to help consumers make healthier food choices. A five-category colour label based on the Food Standards Agency Nutrient profiling system (FSA score) has been proposed in France to be implemented on the front-of-pack of foods (the five-colour nutrition label - 5-CNL). Objectives were to evaluate the ability of the 5-CNL to discriminate nutritional quality between types of breakfast cereals, within a category and in equivalent products, as well as its ability to change through product reformulation.

**Methods:**

Nutritional information was collected through an Internet and supermarket research for N = 433 breakfast cereals (N = 380 complete data included in the analyses). Breakfast cereals were categorized according to common attributes in terms of processing and/or ingredients used. The FSA score and 5-CNL category allocation were computed for each cereal. Nutrient content and FSA score were compared across types of cereals. Distribution within the 5-CNL categories was assessed across types of cereals and for equivalent products. Impact of reformulation (reduction of 5 and 10% in simple sugar, saturated fat and sodium) on the 5-CNL category allocation was compared to original allocation with Bapkhar’s tests of homogeneity of marginal distribution.

**Results:**

Variability in nutritional quality of breakfast cereals was high, as reflected by the FSA score (range −7- 22 for a theoretical range of −15-40) and the 5-CNL (all five categories represented). The 5-CNL allowed for discrimination across types of cereals, within categories of breakfast cereals and for equivalent products (at least 3 categories of the 5-CNL represented). Reformulation scenarios allowed for significant change in 5-CNL allocation: 5% reduction in sugar lead to a modification of the label for 4.21% of products while a reduction of 10% of sugar, saturated fat and sodium lead to a modification of the label for 19.2% of products.

**Conclusion:**

The 5-CNL adequately discriminates between breakfast cereals. It would therefore be an adequate tool for consumer information on nutritional quality of foods in the French context.

**Electronic supplementary material:**

The online version of this article (doi:10.1186/s12889-015-1522-y) contains supplementary material, which is available to authorized users.

## Background

In France, 75.2% of children and 84.9% of adults systematically have breakfast [1,2]. Moreover, 16.8% of adults and 60.4% of children are breakfast cereals consumers [1,2]. Breakfast cereals are therefore significant contributors to daily energy and nutrient intake [3-7].

However, nutritional quality of breakfast cereals is variable [8-10]. They can be considered as highly processed [11], and those marketed to children have regularly been found to have higher contents in sugar than those marketed for adults [12]. Given current knowledge as to content in sugar of breakfast cereals, parents are cautioned against excessive intake of sweet cereals for children [13]. However, currently no specific mean is given as how to distinguish ‘sweet’ cereals from healthier choices. Available information to do so currently includes advertisement or nutritional labelling on food packages [14].

Current legislation in the EU regulates nutritional labelling, with mandatory information on content (per 100 g) in energy, carbohydrates, simple sugars, fat, saturated fat, proteins and sodium, and fibres as optional [15]. Nutritional values are given at the back of every package, but are regarded as difficult to understand, especially for subjects with low educational level [16,17]. To help consumer information, a voluntary complementary nutritional information label can be added at the front-of-pack [15]. Some manufacturers have already developed their own, often giving information on content in several nutrients (energy, sugars, saturated fat an sodium), based on the percentage of Guidelines for Daily Amounts (GDA). However, no single simplified format has been implemented in France [18]. The EU regulation leaves the possibility for each European country to develop its own nutritional information label, and to apply this single format to the entire food supply [15]. Nutrient profiling systems can be viewed as potential support tools for such a simplified nutritional information label. They aim at positioning individual foodstuffs based on their nutritional characteristics [19-21], by giving a general assessment of the ‘nutritional quality’ of a given food or beverage, taking into account current knowledge in nutrition and health relationships.

Multiple nutrient profiling systems have been developed in the world [19,20,22], with varying degrees of validity [23-29]. They usually take into account content in energy, macronutrients and micronutrients of foods, balancing between ‘unhealthy’ components (such as saturated fat or added sugar) and ‘healthy’ components (such as vitamins and minerals). Computation leads to a single global score of the nutritional quality of the food. Subsequently, the score can be used either as a continuous score, or as categorical. Cut-offs need then to be defined, which can lead to dichotomous ‘healthy’ and ‘less healthy’ foods, or to multiple categories. Among nutrient profiling systems having been developed in Europe, some are currently in use for food labeling (namely the Green Keyhole [30] and Choices [31]) or for regulation of advertising to children (the Food Standards Agency (FSA) nutrient profiling system [23,32,33]). One the most scientifically validated nutrient profiling systems in the European context is the FSA nutrient profiling system [23,32,33].

Public health authorities in France are currently examining the opportunity of introducing a comprehensive and simplified nutritional information label on foodstuff, based on the FSA score and including five different categories of nutritional quality [34]. The adoption of a simplified nutrition label is the object of a law, which should be reviewed by the Parliament in the first semester of 2015. This simplified nutritional information system would appear on the front-of-package of every foodstuff and would be colour-coded with five colours from ‘green’ (highest nutritional quality) to ‘red’ (lowest nutritional quality). The five categories would be presented in the form of a chain of five discs of the different colours (Green/yellow/orange/pink/red), with a larger disc representing the nutritional quality of the product (see Additional file 1: Table S1). Corresponding letters from A to E would be added in each disc.

Recent research data tends to confirm the possible use of the FSA score in a five-category classification of foods [35], however, data on application of such a scheme to the actual food supply in France is inexistent.

Our objective was to investigate the ability of a five-category system for nutritional information to discriminate nutritional quality of foodstuffs in the French context. Breakfast cereals were used as an exemplary case for this system. Performance was investigated according to the objectives formulated for the nutritional information label: 1) Discrimination between categories of cereals and within a category of cereals; 2) Discrimination between apparently equivalent products and 3) Potential change in the label category according to reformulation scenarios.

## Methods

### Data collection

Nutritional information on breakfast cereals was collected by trained dieticians from Internet websites from April, 1^st^, 2014 to October 1^st^, 2014. Corporate brand sites, online supermarkets and consumers’ nutritional information websites were visited. Data collection was completed with an additional supermarket research from three different sites in Paris, representing three major food retailers (Simply (Auchan chain), Casino, Carrefour). Some common references from supermarkets and Internet websites were checked for consistency, and duplicates were removed. Supermarket data was used mainly to complete nutritional information from websites.

For each product, brand name, commercial product name and nutrition labelling information at the back of the package were recorded. Energy per 100 g was recalculated from information on content in carbohydrate, fat and proteins.

Internet research obtained nutritional data for 402 references (327 with complete nutritional data for the computation of the FSA score and 75 with incomplete data). Additional supermarket data collection retrieved complete nutritional information for 22 existing Internet references and added 31 complete references. A total of 380 (87.8% of total sample) references with complete nutritional data were used in the analyses.

### Classification of products

Breakfast cereals were classified according to the type of cereals, taking into account common attributes in terms of processing and/or ingredients used. Classification was made using product name and allegation, as follows: chocolate-flavoured cereals (e.g. chocolate flavoured wheat flakes, Nestle’s Chocapic®), honey/caramel cereals (e.g. honey puffed wheat cereals, Kellogg’s Honey Smacks®), light cereals (e.g. cereals marketed for dieting subjects and/or whole-grain cereals, Kellogg’s Special K®), muesli flakes (e.g. mixed granola including rolled cereals such as oats, cornflakes, wheat or rye flakes, Kellogg’s Granola®), crunchy mueslis (e.g. mueslis with an additional bakery process, Jordan’s Country Crisps®), oat flakes (e.g. Quaker’s Oat Flakes®), cornflakes/other plain cereals (e.g. Kellogg’s Cornflakes®), fibre-rich flakes (e.g. cereals marketing their richness in fibres, All Bran®), whole wheat cereals (bite-size, e.g. Weetabix®) and filled cereals (bite-size cereals filled with chocolate, Kellogg’s Krave®). Classification used was similar to the one used by the French Observatory of Food Quality (OQALI), for comparison purposes [9]. Whenever possible, matching products from different brands were identified for each type of cereals, taking into account description of the product and final aspect (e.g. chocolate flavoured wheat flakes, similar to Kellogg’s Chocapic®). These products are hereafter termed ‘equivalent products’. Classification according to type of brand included three categories: national brands, store brands and discount brands. Organic and regular products were also identified.

### Nutrient profiling system and labelling category allocation

For each product, the FSA score was computed taking into account nutrient content for 100 g. It allocates positive points (0–10) for content in energy (KJ), total sugar (g), saturated fatty acids (g) and sodium (mg). Negative points (0–5) are allocated to content in fruits, vegetables and nuts, fibers and proteins. Final score is based on a discrete continuous scale from −15 (most healthy) to +40 (less healthy) (see Additional file 1: Table S1).

Products were then classified in five categories. The statistical quintiles of the FSA score observed in the nutritional composition database of the Nutrinet-santé study were used as cut-offs [35]. This published database reflects foods usually consumed in the French diet. These categories were used to define the nutritional information labelling, as the following colours [35]: ‘Green’ (−15 to −2), ‘Yellow’ (−1 to 3), ‘Orange’ (4 to 11), ‘Pink’ (12 to 16) and ‘Red’ (17 and above). This categorization is hereafter termed Five-colour nutritional information label or ‘5-CNL’ (see Additional file 1: Table S1). Products were also categorized taking into account the British OfCom cut-off for ‘Healthy’ and ‘Less healthy’ foods (FSA score ≤4 for ‘Healthy’ and >4 for ‘Less healthy’ foods) [36,37].

### Statistical analysis

Median and interquartile range (IQR) of the nutrients accounted for in the score (energy, simple sugars, saturated fat, sodium, proteins and fibres), total fat and carbohydrates and total FSA score were compared across types of cereals, using the non-parametric Kruskall-Wallis tests. The 5-CNL categorization was compared across types of cereals by a chi-square test. Discriminating performance of the 5-CNL within types of cereals and for equivalent products (whenever more than 10 products were considered equivalent) was assessed. Number of categories represented within each type of cereals and for equivalent products was considered as indicators of discriminating performance of the 5-CNL. Discriminating performance was deemed high if at least three categories were represented.

Reformulation scenarios included reductions by 5 or 10% for sugar, saturated fat and sodium, respectively, both alone and in combination. Reformulation scenarios were chosen based on observed reformulation objectives made by manufacturers in the framework of the PNNS [38,39]. The impact of such reformulation on the 5-CNL was compared with original composition with Bapkhar’s tests of homogeneity of marginal distribution.

All tests were two-sided and a P value < 0.05 was considered significant. Statistical analyses were performed using SAS® software (9.3 version, Cary, NC, USA).

## Results

The most represented type was crunchy mueslis (N = 99, 26.1% of total), followed by chocolate-flavoured cereals (N = 89, 23.4% of total), light cereals (N = 60, 15.8% of total), filled cereals (N = 40, 10.5% of total) and honey/caramel sweet cereals (N = 35, 9.2% of total) (Table 1).

**Table 1:** **Characteristics of the sample of breakfast cereals in the French market**, **2014** (**N** = **380**)

	**N**	%
Type of cereals		
Crunchy muesli	99	26.05
Chocolate-flavoured cereals	89	23.42
Light cereals	60	15.79
Filled cereals	40	10.53
Honey/caramel sweet cereals	35	9.21
Cornflakes/other plain cereals	20	5.26
Muesli flakes	14	3.68
Oat flakes	12	3.16
Fibre-rich flakes	11	2.89
Type of brands		
Store brands	179	47.11
Discount brands	27	7.11
National brands	174	45.79
Regular/Organic		
Organic	110	28.95
Regular	270	71.05
Ofcom category		
Healthy (FSA score < =4)	80	21.05
Less healthy (FSA score > 4)	300	78.95

Nutritional content across types of cereals exhibited high variability for all nutrients, but more particularly for simple sugars (P < 0.001 across all categories), sodium (P < 0.001 across all categories) and saturated fat (P < 0.001 across all categories) (Table 2).

**Table 2:** **Nutritional characteristics of breakfast cereals according to type of breakfast cereals (N = 380)**

	**Crunchy mueslis**	**Chocolate**-**flavoured cereals**	**Light cereals**	**Filled cereals**	**Honey**/**caramel sweet cereals**
N	99	89	60	40	35
FSA score	9 (6;13)	8 (7;9)	8 (6;11)	12 (10;15)	10 (7;12)
Kcal	440.5 (410;457.9)	373.7 (370;383)	374.95 (368.8;390)	434.6 (420.5;441.5)	376.9 (372.1;381.1)
Carbohydrates	63.8 (60.3;67)	78 (75;82)	76 (72.4;77.8)	67 (65.7;69.5)	86 (80.1;86)
Simple sugars	23 (20;25.7)	30 (27.69;33)	20 (16.5;23.65)	29 (27.5;32.5)	28 (24.5;32)
Fat	16.2 (10.9;19.1)	2.7 (2.4;4.5)	4.6 (1.5;6.6)	15 (13.1;16)	1.1 (0.9;2.5)
Saturated fat	6.1 (2.8;8)	1.4 (1;1.8)	1.25 (0.3;3.5)	4.1 (3.3;5.6)	0.2 (0.2;0.4)
Sodium	100 (20;222)	190 (120;270)	400 (320;453)	279.5 (200;300)	400 (140;450)
Proteins	8.5 (7.8;9.4)	7.9 (7;8.6)	9.45 (8;11.25)	7.4 (7;8)	6 (5.4;7)
Fibres	7.1 (6;8)	5.5 (4;6.2)	4 (3.5;5.05)	4.75 (4;5.45)	2.8 (2;4)
	**Cornflakes/other plain cereals**	**Muesli flakes**	**Oat flakes**	**Fibre-rich flakes**	**Pvalue**
N	20	14	12	11	
FSA score	8 (3;8.5)	−3.5 (−5;-2)	−3.5 (−5;-0.5)	2 (−4;7)	<0.001
Kcal	374.25 (368.55;378.4)	355.95 (340.4;373.5)	358.65 (343.75;385.6)	367.5 (330.5;383.6)	<0.001
Carbohydrates	82.6 (81;84)	63.15 (58.6;67.7)	58.5 (56.6;65.8)	69 (65;75.7)	<0.001
Simple sugars	7.5 (4.5;8.6)	18.05 (10.6;24.8)	1.9 (1.1;8.8)	18 (4.5;24)	<0.001
Fat	1.05 (1;1.65)	7.35 (5.5;9.3)	7.75 (6.8;9.1)	4 (2.9;6)	<0.001
Saturated fat	0.3 (0.2;0.55)	1.3 (1;1.6)	1.4 (1.1;1.7)	0.7 (0.5;4)	<0.001
Sodium	620 (245;750)	25 (8;100)	7 (1;310)	460 (350;620)	<0.001
Proteins	7.95 (7.6;8)	9.3 (8.1;11.3)	12.4 (10.6;13.85)	11 (8;14)	<0.001
Fibres	3.4 (3.05;3.75)	10.65 (8;12)	10.25 (6.9;12)	10 (9;27)	<0.001

Values are median (interquartile range). P value obtained with Kruskall-Wallis tests across all categories.

**Figure 1:**
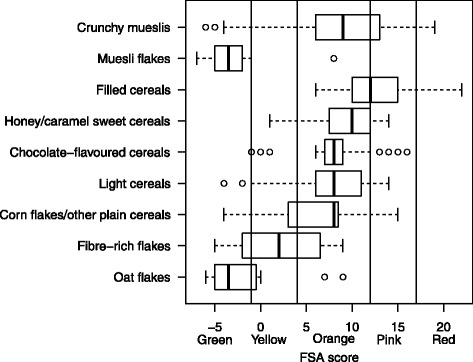
**Distribution of FSA scores across categories of breakfast cereals (N** = **380).** The boundary of the box nearest to the right indicates the 25th percentile, the line within the box marks the median, and the boundary of the box furthest from the right indicates the 75th percentile. Whiskers (error bars) above and below the box indicate the lower limit (25th percentile – 1.5 *(Inter-quartile range) and the upper limit (75th percentile + 1.5 *(Inter-quartile range)). The circles are individual outlier points.

Overall, for breakfast cereals, FSA score ranged from −7 (most healthy, for a fruit muesli flake) to 22 (least healthy, for a chocolate filled cereal). FSA score was highest for filled cereals (median score 12 (IQR = 10;15) and lowest for muesli flakes (median score −3.5 (IQR = 5;-2), P < 0.001 across all categories) (Table 2). Types of breakfast cereals significantly differed in their 5-CNL allocation: the majority of muesli flakes and oat flakes were categorized as ‘Green’, while the majority of chocolate-flavoured cereals and light cereals were categorized as ‘Orange’ (Table 3). For crunchy mueslis and filled cereals, some products were even categorized as ‘Red’ (Table 3). Variability within types was the most important for crunchy mueslis, corn flakes/other plain cereals and was relatively low for chocolate-flavoured cereal and muesli flakes (Figure 1). All types of cereals were distributed in three or more categories of the 5-CNLshowing high discriminating performance of the system (Table 3 and Figure 1). Variability in 5-CNL was also important in equivalent products, as all products were distributed in at least three categories, and even five categories for chocolate crunchy mueslis (Table 4).

**Table 3:** **Distribution across categories of the 5**-**CNL nutritional information system according to type of breakfast cereals (N = 380)**

	**Nutritional information category**					
	**Green**	**Yellow**	**Orange**	**Pink**	**Red**	
	**N (%)**	**N (%)**	**N (%)**	**N (%)**	**N (%)**	**Pvalue**
Crunchy mueslis	11 (11.1)	9 (9.1)	46 (46.5)	27 (27.3)	6 (6.1)	<0.001
Chocolate-flavoured cereals	-	8 (9.0)	75 (84.3)	6 (6.7)	-	
Light cereals	3 (5.0)	7 (11.7)	43 (71.7)	7 (11.7)	-	
Filled cereals	-	-	15 (37.5)	18 (45.0)	7 (17.5)	
Honey/caramel sweet cereals	-	2 (5.7)	23 (65.7)	10 (28.6)	-	
Cornflakes/other plain cereals	4 (20.0)	1 (5.0)	14 (70.0)	1 (5.0)	-	
Muesli flakes	11 (78.6)	2 (14.3)	1 (7.1)	-	-	
Oat flakes	8 (66.7)	2 (16.7)	2 (16.7)	-	-	
Fibre-rich flakes	3 (27.3)	3 (27.3)	5 (45.5)	-	-	

P value obtained from Chi square test.

**Table 4:** **Distribution across categories of the 5**-**CNL nutritional information system for matching products**

	**Nutritional information category**					
	**Green**	**Yellow**	**Orange**	**Pink**	**Red**	
	**N (%)**	**N (%)**	**N (%)**	**N (%)**	**N (%)**	**Total**
Crunchy mueslis						
Chocolate crunchy muesli	1 (2.9)	1 (2.9)	12 (34.3)	17 (48.6)	4 (11.4)	35
Fruit crunchy muesli	9 (24.3)	4 (10.8)	20 (54.1)	4 (10.8)	-	37
Nuts crunchy muesli	9 (60.0)	4 (26.7)	2 (13.3)	-	-	15
Chocolate-flavoured cereals						
Chocolate wheat flakes	1 (4.5)	1 (4.5)	18 (81.8)	2 (9.1)	-	22
Chocolate puffed rice	-	1 (7.7)	10 (76.9)	2 (15.4)	-	13
Chocolate puffed cereal	-	3 (15.0)	17 (85.0)	-		20
Light cereals						
Chocolate light cereals	-	2 (15.4)	9 (69.2)	2 (15.4)	-	13
Fruit light cereals	1 (9.1)	1 (9.1)	9 (81.8)	-	-	11
Unflavoured light cereals	-	1 (11.1)	8 (88.9)	-	-	9
Filled cereals						
Cereals filled with milk chocolate	-	-	3 (33.3)	2 (22.2)	4 (44.4)	9
Cereals filled with chocolate hazelnut	-	-	5 (31.3)	11 (68.8)		16
Honey/caramel sweet cereals						
Honey corn balls	-	1 (9.1)	7 (63.6)	3 (27.3)	-	11
Cornflakes/other plain cereals						
Corn flakes	4 (19.0)	1 (4.8)	15 (71.4)	1 (4.8)	-	21

Breakfast cereals (N = 380).

Reformulation scenarios lead to significant modifications in the 5-CNL allocation (Table 5). Reduction in sugar lead to the most significant modifications (4.21% change in 5-CNL for 5% reduction in sugar alone, P = 0.002) (Table 5). Reduction by 10% for sugar, saturated fat and sodium combined lead to 19.2% of change in the 5-CNL categorization (Table 5).

**Table 5:** **Distribution across categories of the 5**-**CNL and change from baseline distribution according to product reformulation**

	**Nutritional information category**							
	**Green**	**Yellow**	**Orange**	**Pink**	**Red**	**Change**	**Pvalue**	
	**N (%)**	**N (%)**	**N (%)**	**N (%)**	**N (%)**	**N (%)**		**Number of cereals in each category with change in labeling**
Baseline	40 (10.5)	34 (8.9)	224 (58.9)	69 (18.2)	13 (3.4)	-		
−5% Sugar	42 (11.1)	39 (10.3)	223 (58.7)	64 (16.8)	12 (3.2)	16 (4.2)	0.002	Honey/caramel sweet cereals (N = 6), crunchy muesli (N = 3), chocolate-flavoured cereals (N = 2), filled cereals (N = 2), light cereals (N = 2), muesli flakes (N = 1)
−5% Sodium	40 (10.5)	36 (9.5)	223 (58.7)	68 (17.9)	13 (3.4)	3 (0.8)	0.22	Light cereals (N = 1), fibre-riche cereals (N = 1), corn flakes/other plain cereals (N = 1)
−5% Saturated fat	41 (10.8)	34 (8.9)	226 (59.5)	67 (17.6)	12 (3.2)	6 (1.6)	0.19	crunchy muesli (N = 2), filled cereals (N = 2), light cereals (N = 2)
−5% Sugar and Sodium	42 (11.1)	41 (10.8)	221 (58.2)	64 (16.8)	12 (3.2)	18 (4.7)	0.0008	Honey/caramel sweet cereals (N = 6), crunchy muesli (N = 3), chocolate-flavoured cereals (N = 2), filled cereals (N = 2), light cereals (N = 2), muesli flakes (N = 1), fibre-riche cereals (N = 1), corn flakes/other plain cereals (N = 1)
−5% Saturated fat and Sodium	41 (10.8)	36 (9.5)	225 (59.2)	66 (17.4)	12 (3.2)	9 (2.4)	0.0559	filled cereals (N = 3), crunchy muesli (N = 2), light cereals (N = 2), fibre-riche cereals (N = 1), corn flakes/other plain cereals (N = 1)
−5% Saturated fat and Sugar	43 (11.3)	39 (10.3)	225 (59.2)	62 (16.3)	11 (2.9)	22 (5.8)	<0.0001	Honey/caramel sweet cereals (N = 6), crunchy muesli (N = 5), filled cereals (N = 4), light cereals (N = 4), chocolate-flavoured cereals (N = 2), muesli flakes (N = 1)
−5% Saturated fat, Sodium and Sugar	43 (11.3)	41 (10.8)	223 (58.7)	62 (16.3)	11 (2.9)	24 (6.3)	<0.0001	Honey/caramel sweet cereals (N = 6), crunchy muesli (N = 5), filled cereals (N = 4), light cereals (N = 4), chocolate-flavoured cereals (N = 2), muesli flakes (N = 1), fibre-riche cereals (N = 1), corn flakes/other plain cereals (N = 1)
−10% Sugar	43 (11.3)	48 (12.6)	219 (57.6)	61 (16.1)	9 (2.4)	36 (9.5)	<0.0001	Honey/caramel sweet cereals (N = 9), crunchy muesli (N = 8), filled cereals (N = 7), chocolate-flavoured cereals (N = 6), light cereals (N = 4), muesli flakes (N = 1),, corn flakes/other plain cereals (N = 1)
−10% Sodium	40 (10.5)	46 (12.1)	224 (58.9)	59 (15.5)	11 (2.9)	26 (6.8)	<0.0001	Light cereals (N = 1), fibre-riche cereals (N = 1), corn flakes/other plain cereals (N = 1)
−10% Saturated fat	41 (10.8)	37 (9.7)	225 (59.2)	68 (17.9)	9 (2.4)	14 (3.7)	0.0058	crunchy muesli (N = 5), filled cereals (N = 5), light cereals (N = 3), chocolate-flavoured cereals (N = 1)
−10% Sugar and Sodium	43 (11.3)	65 (17.1)	213 (56.1)	50 (13.2)	9 (2.4)	64 (16.8)	<0.0001	Light cereals (N = 14), chocolate-flavoured cereals (N = 13), crunchy muesli (N = 12), filled cereals (N = 10), honey/caramel sweet cereals (N = 10), fibre-riche cereals (N = 2), corn flakes/other plain cereals (N = 2), muesli flakes (N = 1)
−10% Saturated fat and Sodium	41 (10.8)	49 (12.9)	226 (59.5)	56 (14.7)	8 (2.1)	40 (10.5)	<0.0001	filled cereals (N = 9), light cereals (N = 9), crunchy muesli (N = 8), chocolate-flavoured cereals (N = 5), honey/caramel sweet cereals (N = 5), fibre-riche cereals (N = 2), corn flakes/other plain cereals (N = 2)
−10% Saturated fat and Sugar	44 (11.6)	51 (13.4)	221 (58.2)	57 (15)	7 (1.8)	49 (12.9)	<0.0001	crunchy muesli (N = 14), filled cereals (N = 10), honey/caramel sweet cereals (N = 9), chocolate-flavoured cereals (N = 8), light cereals (N = 6), corn flakes/other plain cereals (N = 1), muesli flakes (N = 1)
−10% Saturated fat, Sodium and Sugar	44 (11.6)	68 (17.9)	210 (55.3)	52 (13.7)	6 (1.6)	73 (19.2)	<0.0001	Light cereals (N = 16), crunchy muesli (N = 16), chocolate-flavoured cereals (N = 15), filled cereals (N = 11), honey/caramel sweet cereals (N = 10), fibre-riche cereals (N = 2), corn flakes/other plain cereals (N = 2), muesli flakes (N = 1)

Breakfast cereals (N = 380).

P value obtained with Bapkhar’s tests.

## Discussion

Our results show that through the example of breakfast cereals, a five-category nutritional information label (the 5-CNL) based on the FSA nutrient profiling system displays high performance to discriminate nutritional quality across types of breakfast cereals, within a category of breakfast cereals, within equivalent products. Moreover, reformulation would allow significant modifications in 5-CNL category allocation.

Our study used breakfast cereals as an exemplary case for the application of the 5-CNL in the actual French market. However, data suggests that nutritional variability in most groups of foods is very large, and that our results would be reproducible in the French market at large [35].

One of the strengths of our study is the high number of data collected. Our database included 427 products, though all did not have complete information. As a comparison, the French OQALI database included 449 products in 2011, corresponding to 74.6% of the market in volume [40]. However, public OQALI data do not contain information on specific products, therefore not allowing for a direct comparison between apparently equivalent product, as we did. Although we did not collect data on market volume share, a high degree of overlap between the two databases can be expected, and our data could therefore be considered as representative of the whole breakfast cereals market in France.

Our study is subject to some limitations. First, the majority of data were collected through Internet search. While company websites gave reliable and complete information for the majority of their products, information was less detailed for other types of brands. Moreover, while we have been able to check consistency for some common references in both Internet and supermarket search, we were not able to test reliability of web sources, or thoroughly compare web to supermarket sources, and some data might have been incorrect. However, data collection was performed by trained dieticians, who were able to identify implausible nutritional data. Second, we were not able to take into account market share of each product, which would have strengthened our analyses concerning the impact of reformulation. Third, we limited our analyses on breakfast cereals only as an exemplary case of the application of the FSA score to foods. A more generalized investigation of food products currently on the French market would allow for the drawing of more definite conclusions as to its potential as a front-of-pack nutritional labelling system. Finally, we used cut-offs obtained from an analysis using the French Nutrinet-Study food composition data, which reflects foods consumed in France, but not the French food market. Modification of the cut-offs might modify some results. However, as distribution in the continuous FSA score appeared homogenous, effect of the modification of the cut-off would be of limited impact.

Variability in nutritional composition of breakfast cereals observed in our sample, both across cereal type and within a type are in line with results from the OQALI [9]. As in our study, variability in nutritional composition was higher for crunchy mueslis compared with chocolate-flavoured cereals [9]. However, the study from Goglia et al. took into account nutrients one by one, and did not provide a comprehensive validated estimate of nutritional quality of breakfast cereals. Our results suggest that the variability of nutritional composition observed for each nutrient is adequately reflected in the FSA score.

The FSA score was initially designed to categorize foods in two categories: ‘Healthy’ (for foods with FSA scores <4) and ‘Less healthy’ (for foods with FSA scores ≥4) [37,41]. Such classification has been validated in the British food environment [23] and in the New Zealand food environment [42]. Using this dichotomized tool, Devi et al. were able to identify differences in nutritional quality across types of cereals [8]. On the whole, 74% of breakfast cereals were considered as ‘Healthy’, the percentage ranging from 42% for ‘cereals for kids’ to 100% for ‘oats’ [8]. As the ‘Healthy’ category corresponds to the ‘Green’ and ‘Yellow’ categories combined, such dichotomization would allow for some discrimination across types of cereals in our sample, or even within a category, but would not be so efficient in discriminating the nutritional quality of equivalent products: for example, all chocolate filled cereals would be categorized as ‘less healthy’. Use of multiple categories ensures a higher discrimination performance of the 5-CNL. Moreover, some have argued that the use of binary scores tends to induce the idea of ‘good’ and ‘bad’ foods, promoting dichotomous thinking [43].

Research on front-of-package nutrition labelling suggests that simple nutrition formats, giving a single global estimate of the nutritional quality of the food are more easily understood by consumers, more particularly among subjects with lower educational levels [44-46]. Moreover, they appear to be more appropriate for use in real purchase situations as they are fast to identify and understand by consumers [47]. The simple format of the 5-CNL would therefore be another argument of its performance.

Cereal reformulation, and more generally, improvement in food supply can be regarded as a public health initiative, as it can lead to significant improvement in nutrient intake of the population [48]. The French public health nutrition program (Programme National Nutrition Santé, PNNS), aims also at improving quality of the food supply, by signed charters of improvements in nutritional quality with manufacturers. However, the monitoring of nutritional quality of breakfast cereals in France between 2008 and 2011 showed significant improvements in sugars only for filled cereal (−7%) and crunchy mueslis (−10%); in sodium only in light cereals (−28%); and no improvement of fat content [40].

Foodstuff labelling can entice manufacturers to reformulate their products [17]. Impact can be differential depending on the type of labelling information displayed [17]. Indeed, while introduction of the ‘Daily Intake Guide’ on Australian breakfast cereals did not lead to any significant change in nutritional quality of breakfast cereals [10], the ‘Pick the tick’ in new Zealand led to an average 61% reduction in salt content of breakfast cereals [49]. Format of the label could in part explain such contrasting results: the ‘Daily Intake Guideline’ label gave complex nutrient-by-nutrient information similar to the GDAs, while the ‘Pick the tick’ label was a single label indicating the product as ‘healthy’. Our results suggest that the implementation of the 5-CNL in France could lead to substantial product reformulation, as even minor reformulations would lead to significant modifications in 5-CNL categorization.

## Conclusion

Our study supports a five-category nutritional information label to discriminate between breakfast cereals. The 5-CNL would therefore be a useful tool to rapidly and easily inform consumer information about nutritional quality of foods and to stimulate product reformulation by manufacturers in the French context.

## Additional file

Additional file 1: Table S1. FSA score computation and CNL5 labeling attribution.

## Abbreviations

FSA: Food Standards Agency
OfCom: Office of Communication
PNNS: Programme National Nutrition Santé
5-CNL: Five-colour nutritional information label
