# Supplementary material for: Performance of a five category front-of-pack labelling system – the 5-colour nutrition label – to differentiate nutritional quality of breakfast cereals in France
Source: BMC Public Health. 2015 Feb 25;15:179. doi: 10.1186/s12889-015-1522-y (PMC4349460; doi:10.1186/s12889-015-1522-y)
Supplement: Additional file 1: Table S1. — FSA score computation and CNL5 labeling attribution. [file 12889_2015_1522_MOESM1_ESM.doc]

Points are allocated according to the nutrient content for 100g of foods or beverages.

Points are allocated for ‘Negative’ nutrients (A points) and can be balanced according to ‘Positive’ nutrients (C points). Final computation depends on the level of A points and content in fruits, vegetables and nuts of the product

**A points**

Total A points = (points for energy) + (points for saturated fat) + (points for total sugar) + (points for sodium)

| ***Points*** | **Energy (kJ)** | **Sat Fat (g)** | **Total Sugar (g)** | **Sodium (mg)** |
| --- | --- | --- | --- | --- |
| 0 | ≤ 335 | ≤ 1 | ≤ 4.5 | ≤ 90 |
| 1 | >335 | >1 | >4.5 | >90 |
| 2 | >670 | >2 | >9 | >180 |
| 3 | >1005 | >3 | >13.5 | >270 |
| 4 | >1340 | >4 | >18 | >360 |
| 5 | >1675 | >5 | >22.5 | >450 |
| 6 | >2010 | >6 | >27 | >540 |
| 7 | >2345 | >7 | >31 | >630 |
| 8 | >2680 | >8 | >36 | >720 |
| 9 | >3015 | >9 | >40 | >810 |
| 10 | >3350 | >10 | >45 | >900 |

**C points**

Total C points = (points for fruit, vegetables and nuts) + (points for fiber) + (points for proteins)

| ***Points*** | **Fruit, Veg & Nuts (%)** | **Fiber (g) *** | **Protein (g)** |
| --- | --- | --- | --- |
| 0 | ≤ 40 | ≤ 0.7 | ≤ 1.6 |
| 1 | >40 | >0.7 | >1.6 |
| 2 | >60 | >1.4 | >3.2 |
| 3 | - | >2.1 | >4.8 |
| 4 | - | >2.8 | >6.4 |
| 5 | >80 | >3.5 | >8.0 |

*FSA score allocates different thresholds for fibers, depending on the measurement method used. We used NSP cut-offs to compute fibers score

**Global score computation**

- If Total A points <11, then FSA score =Total A points – Total C score
- If Total A points ≥11,
  - If points for fruits, vegetables and nuts =5, then FSA score =Total A points – Total C score
  - Else if points for fruits, vegetables and nuts <5, then FSA score = Total A points – (points for fiber + points for fruit, vegetables and nuts)

**CNL5 labeling colour attribution**

| **Global score** | **Category** | **Label** |
| --- | --- | --- |
| -15 – -2 | Green | **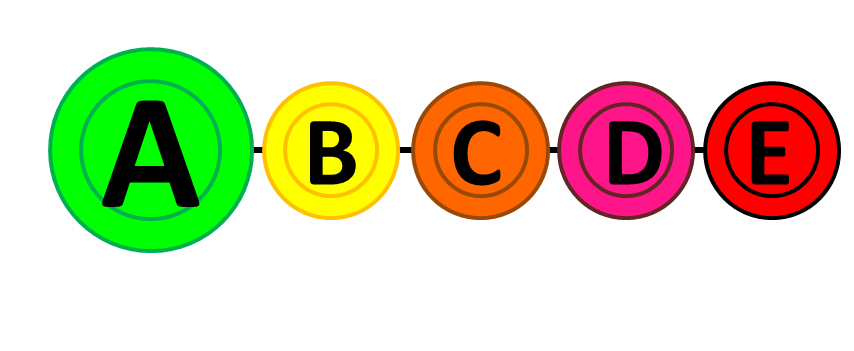** |
| -1 – 3 | Yellow | **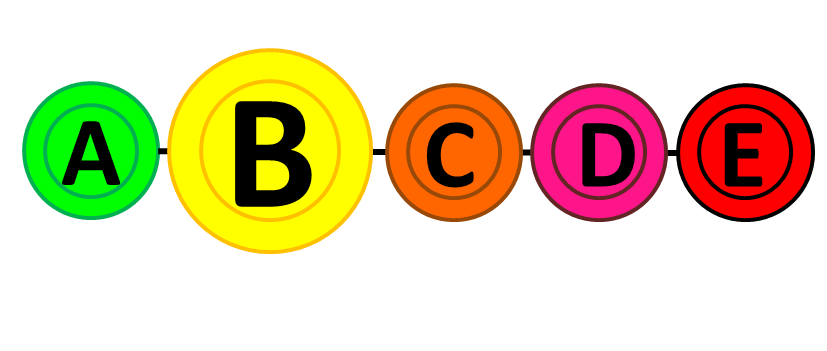** |
| 4 – 11 | Orange | **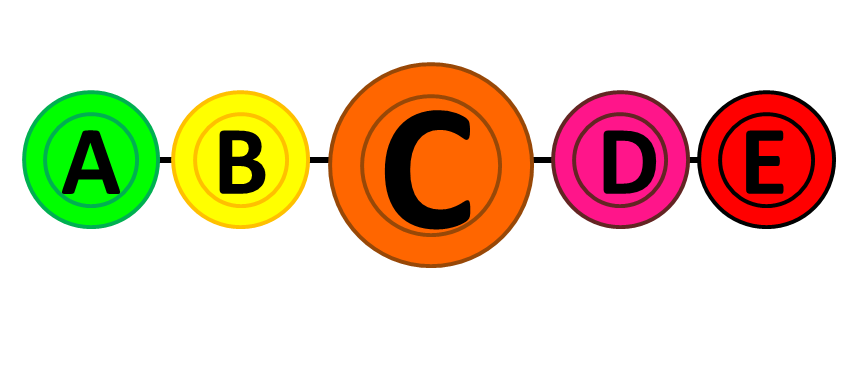** |
| 12 – 16 | Pink | **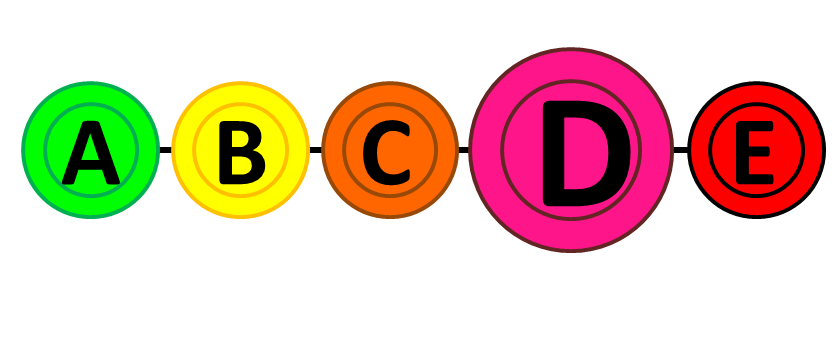** |
| 17 – 40 | Red | **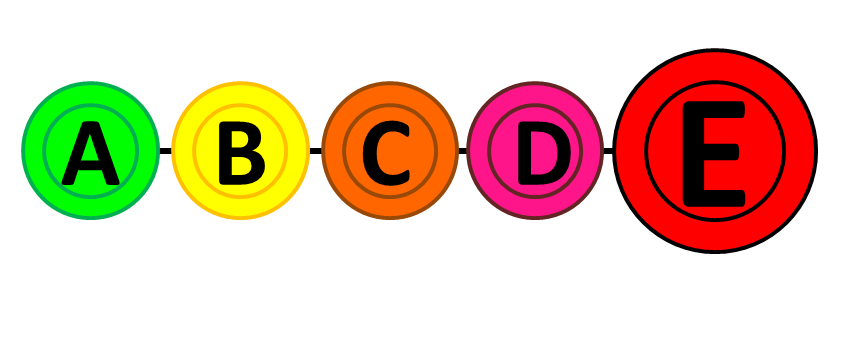** |
